# Immune Modulation as an Effective Adjunct Post-exposure Therapeutic for *B*. *pseudomallei*

**DOI:** 10.1371/journal.pntd.0005065

**Published:** 2016-10-28

**Authors:** William J. Wilson, Maryam F. Afzali, Jason E. Cummings, Marie E. Legare, Ronald B. Tjalkens, Christopher P. Allen, Richard A. Slayden, William H. Hanneman

**Affiliations:** 1 Center for Environmental Medicine, Colorado State University, Fort Collins, Colorado, United States of America; 2 Environmental & Radiological Health Sciences, Colorado State University, Fort Collins, Colorado, United States of America; 3 Department of Microbiology, Immunology and Pathology, Colorado State University, Fort Collins, Colorado, United States of America; University of Tennessee, UNITED STATES

## Abstract

Melioidosis is caused by the facultative intracellular bacterium *Burkholderia pseudomallei* and is potentially fatal. Despite a growing global burden and high fatality rate, little is known about the disease. Recent studies demonstrate that cyclooxygenase-2 (COX-2) inhibition is an effective post-exposure therapeutic for pulmonary melioidosis, which works by inhibiting the production of prostaglandin E2 (PGE2). This treatment, while effective, was conducted using an experimental COX-2 inhibitor that is not approved for human or animal use. Therefore, an alternative COX-2 inhibitor needs to be identified for further studies. Tolfenamic acid (TA) is a non-steroidal anti-inflammatory drug (NSAID) COX-2 inhibitor marketed outside of the United States for the treatment of migraines. While this drug was developed for COX-2 inhibition, it has been found to modulate other aspects of inflammation as well. In this study, we used RAW 264.7 cells infected with B pseudomallei to analyze the effect of TA on cell survival, PGE2 production and regulation of COX-2 and nuclear factor- kappaB (NF-ĸB) protein expression. To evaluate the effectiveness of post-exposure treatment with TA, results were compared to Ceftazidime (CZ) treatments alone and the co-treatment of TA with a sub-therapeutic treatment of CZ determined in a study of BALB/c mice. Results revealed an increase in cell viability *in vitro* with TA and were able to reduce both COX-2 expression and PGE2 production while also decreasing NF-ĸB activation during infection. Co-treatment of orally administered TA and a sub-therapeutic treatment of CZ significantly increased survival outcome and cleared the bacterial load within organ tissue. Additionally, we demonstrated that post-exposure TA treatment with sub-therapeutic CZ is effective to treat melioidosis in BALB/c mice.

## Author Summary

*Burkholderia pseudomallei* is the causative agent of melioidosis, a fatal tropical disease endemic in parts of Southwest Asia and Northern Australia. While it was once believed to be isolated to these areas, recent research indicates the global burden on melioidosis is growing. Furthermore, treatment of melioidosis is difficult because of the high occurrence of disease relapse and increasing antibacterial resistance. Recent research suggests that immunomodulation via COX-2 inhibition to subsequently reduce with PGE2 production is an effective therapeutic strategy for melioidosis. The current study was built on this immunomodulatory principle by using an orally administered COX-2 inhibitor and evaluating its effects on the COX-2 and NF-ĸB pathways. We also investigated whether the conjunctive therapies of immunomodulation and antibiotics increased efficacy of the treatment. We confirmed immunomodulation is effective as a post-exposure therapeutic in BALB/c mice. More importantly, we found that conjunctive post-exposure treatment via immunomodulation increased antibacterial treatment efficacy. Conjunctive therapy may prove efficacious for other infectious diseases resembling melioidosis. Hence, further research is needed to identify the long-term effects of the described treatment(s) across multiple animal models.

## Introduction

Melioidosis is a tropical and often fatal disease caused by the aerobic, Gram-negative facultative intracellular bacterium *Burkholderia pseudomallei* [1]. Traditional *B*. *pseudomallei* infection is associated with environmental exposures during the monsoon season in the tropics. It has also been identified as a Tier 1 select agent due to its high mortality rate, ability to cause respiratory infection and its drug resistance. *B*. *pseudomallei* is most common in Southeast Asia and Northern Australia were it is found naturally as a soil-dwelling bacteria [2] [3], Evidence suggests the incidence of melioidosis is underreported and the global burden is increasing with an estimated 169,000 cases per year and 89,000 deaths across 34 countries annually [4]. Therefore, the need to establish multiple therapeutic strategies is paramount.

*B*. *pseudomallei* is naturally resistant to many antibiotics, such as penicillin, many cephalosporins and aminoglycosides, but can be susceptible to such antibiotics as doxycycline, ceftazidime and chloramphenicol. The typical treatment regime for melioidosis lasts 20 weeks with both intravenous and oral phases of antibiotic administration. Due to these intense treatment requirements and antibacterial resistant isolates, relapse of the disease is common [2]. Recent evidence suggests that modulating the immune modulation by inhibiting cyclooxygenase-2 (COX-2) to reduce prostaglandin E2 (PGE2) expression is an effective post-exposure therapeutic. Another consideration of the current work is use of the COX-2 inhibition standard, NS-398, since is not approved for human use [5]. A COX-2 inhibitor with similar effects as NS-398 in a form administered easily to patients would be one step closer to developing a successful immune modulation regimen to treat melioidosis. This has profound implications as effective immune modulation treatment can reduce the selective pressure for bacteria to evolve to become drug resistant. Immune modulation can augment treatment of disease by either enhancing the effectiveness of a given antibiotic or by reducing the antibacterial dose required for treatment. Such interventions are promising developments toward the ultimate goal of eliminating an infectious disease by optimizing the host innate immune response [6].

Tolfenamic acid (TA) belongs to the fenamate class of NSAIDS and can be administered orally and intravenously to various animal species. Although not approved for human use in the United States, oral administration is used elsewhere in the world for treatment of migraines [7] [8]. While TA is primary known for its ability to inhibit COX-2, TA has also shown to be effective at modulating other key players in inflammation such as NF-ĸB. Evidence suggests a reduction in cytoplasmic NF-ĸB p65 activation in colon cancer cells and LPS stimulated RAW 264.7 cells as well [9] [10]. In veterinary medicine, TA has been shown to be a potent inhibitor of NF-ĸB p65 canine-derived tumor cells [11]. With its wide range of inflammatory modulation implications, TA could prove to be a valuable augmentation to current treatment therapeutics for melioidosis. Additionally, because of the broad impact of TA on many inflammatory mediators, its use may further elucidate how *B*. *pseudomallei* causes mortality.

In the present work, COX-2 inhibition was used to reduce the inflammatory response caused by *B*. *pseudomallei*. We examined the role of NF-ĸB, COX-2 and PGE2 during acute pulmonary infection with *B*. *pseudomallei*. For the first specific aim, we investigated the characteristics of the immune response and the potential of TA treatment to modulate the immune response and survival outcome. This work was done *in-vitro* by infecting RAW 264.7 cells with the *B*. *pseudomallei* 1026b Δ*purM* strain Bp82. These results served as the basis of a second specific aim to expand our BALB/c *in-vivo* study protocol and monitor the bacterial dissemination and organ system burden of *B*. *pseudomallei* 1026b in order to confirm the relationship between bacterial burden and dissemination. From there, the study focused on how treatment with TA and known effective antibiotics, alone or in combination, affect survival outcome over time.

## Materials and Methods

### 2.1. Reagents, NSAIDS and Antibodies:

Dimethyl sulfoxide (DMSO) was purchased from ATCC (Manassas, VA). Dulbecco’s Modified Eagles Medium (DMEM) and trypsin used for cell culture were purchased from GE Healthcare Sciences (Hyclone) (Logan, UT) and fetal bovine serum plus (FBS +) was purchased from Atlas Biologicals (Fort Collins, CO). Tolfenamic acid was purchased from Cayman Chemical (Ann Arbor, MI). NF-ĸB monoclonal antibodies where purchased from Santa Cruz Biotechnology (Paso Robles, CA) and the secondary alexa fluor antibodies, 647 and HRP secondary antibodies used for all applications were purchased from Cell Signaling Technologies (Danves, MA). Luria-Bertani (LB) agar, cation adjusted Mueller-Hinton broth (ca-MHB) and COX-2 monoclonal antibodies were purchased from BD Sciences (Franklin Lakes, NJ). Dibutylhydroxytoluene (BHT), bovine serum albumin (BSA), Ceftazidime (CZ), crystal violet (CV) and sodium dodecyl sulfate (SDS) were purchased from Sigma-Aldrich (St. Louis, MO). Formalin and triton X-100 was purchased from ThermoFisher-Scientific (Waltham, MA). Ketamine for animal studies was purchased from Aurora Veterinary Supply (Aurora, CO).

### 2.2. Cell and Bacterial Culture:

RAW 264.7 cells were purchased from ATCC and maintenance was performed to the company’s specifications. Cells were grown in DMEM with 4 mM L-glutamine, 4500 mg/L glucose, 5 mM sodium pyruvate, 1500 mg/L sodium bicarbonate, and 10% FBS. Cells were grown in an incubator at 37°C with 5% CO_2_.

Bp82, a Δ*purM B*. *pseudomallei* 1026b mutant incapable of adenine and thymine biosynthesis, [12] was used as a 1026b BSL-2 surrogate organism for *in-vitro* experiments. 1026b [13] was used for all BSL-3 animal studies. Both strains were prepared by growing 1 colony of the respective bacterial cultures in 50 ml of LB broth for 48 hours at 37°C. Bacterial stock was then frozen back in ~1.0 ml in LB broth and 10% glycol.

### 2.3 Effect of TA on Cytotoxicity:

RAW 264.7 cells were plated in a 96-well plate with 100,000 cells per well and incubated for 24 hours. Wells were washed with sterile PBS, pretreated with either 100μM TA or 0.01% DMSO for 30 minutes, and infected with Bp82 at an MOI of 5. Each time point also included an untreated/infected control and an uninfected/untreated control. At each time point, 100μL of 10% formalin in methanol was added to each treatment well and placed on a rocker for 15 minutes with 20 tilts per minute. The formalin solution was removed and 100μL of a 0.5% solution of CV in 25% methanol/75% ddH2O was added to each well and placed on the rocker for 15 minutes. After staining, the 96 well plates were gently washed under tap water until the water ran clear from each well. The plate was air dried overnight until there was no visible liquid in any well. Finally, the CV was re-suspended from the cells by adding 100μL of 1% SDS solution to each well and placed on the rocker for 30 minutes at room temperature. The plate was then read on a plate reader at 570nm and 600nm. This procedure was adapted and optimized from Kursheed et al [14] and Castro-Garza et al [15]. Each treatment had a technical replicate of 10 and the experiment was run in biological triplicate for statistical significance.

### 2.4 Minimum Inhibitory Concentration (MIC) Determination:

To determine if TA possessed any natural antibiotic properties, a MIC was established. The procedure outlined in [16] was used. Briefly, Bp82 stock was incubated in LB broth overnight (18 hours) at 37°C, passed at a 1:100 dilution, and incubated for an additional 6 hours. This stock was then diluted to reach an optical density of 0.1 at 600 nm in caMHB. This diluted culture was further diluted 1:100 to achieve approximately 1x 10^6^ CFU/ml inoculum. In 50μl of caMHB, 1:2 serial dilutions of TA and CZ were prepared in a 96-well plate with the highest concentration being 128μg/ml to the lowest concentration of 0.0625μg/ml. Each treatment was run in triplicate and inspected by different personnel for verification. 50μl of inoculum was added to each well of the 96-well plate and plate incubated overnight at 37°C for 18 hours. The MIC concentration was determined as the lowest concentration of treatment that resulted in no bacterial growth.

### 2.5 Effect of Treatment on COX-2 and NF-ĸB

RAW 264.7 cells were cultured in six-well plates at a density of 2x10^6^ cells per well for 24 hours. Cells were then pre-treated with 100μM TA or 0.01% DMSO for 30 minutes. The untreated and uninfected control were pretreated with fresh media. After pre-treatment, cells were infected at an MOI of 5, and centrifuged for 5 minutes at 500 rpm to allow the infection to reach the monolayer. At 90 minutes or 6 hours post infection (NF-ĸB/COX-2) cells were washed twice with sterile PBS (pH7.4) and removed utilizing 0.025% trypsin and neutralized with complete DMEM. Cells were fixed in 3.7% paraformaldehyde in PBS for 10 minutes and washed with PBS. Cell membranes were permeabilized with 0.01% triton X-100 in PBS for 10 minutes and the primary antibody stain was added at a dilution of 1:100. Samples were incubated at 42°C for 20 minutes, washed with PBS and re-suspended in 0.01% triton X-100 in PBS and the secondary antibody at a dilution of 1:100. Samples were washed and prepared for analysis in PBS. Flow cytometry was conducted on a Beckman Coulter CyAn ^ADP^ Flow Cytometer operating Summit v4.3 software for data collection. All further data analysis was done with FlowJo software. Samples were run in biological triplicate with two technical duplicates.

### 2.6 Effect of Treatment on PGE2:

RAW 264.7 cells were cultured as described in 2.5. At each time point after infection, the supernatant was removed and 1% BHT solution was added to avoid the free-radical peroxidation as explained in [17]. Samples were then analyzed or stored at -20°C for later analysis. PGE2 analysis and quantification was conducted via ELISA using a PGE2 analysis kit (R&D Systems) in accordance with the manufacturer's instructions, except samples were not diluted as indicated. This experiment was conducted three independent experiments run in duplicate.

### 2.7 Animal Studies

Ethics Statement: Animal experiments were performed in accordance with the recommendations in the Guide for the Care and Use of Laboratory Animals of the National Institutes of Health. The protocol was approved by the Colorado State University Institutional Animal Care and Use Committee, protocol number 15-6138A. Six to eight week old, female BALB/c mice (Jackson Laboratories) were maintained under pathogen-free conditions and allowed free access to sterile food and water with 12 hour light/dark cycles. 45 mice were separated into 9 groups (n = 5/group) depending on treatment and survival regime.

For bacterial challenge, mice were anesthetized with ketamine/xylazine (100/10 mg/kg). The 1026b bacterial inoculum contained ~2×10^3^ CFU suspended in 20 μl sterile saline and was delivered dropwise via pipet. Bacterial CFU were confirmed by plating the inoculum on LB agar. Treatments were initiated 3 hours post infection and repeated every 24 hours for two consecutive days as done by Asakrah et al [5]. Mice were euthanized when morbidity characteristics of hunched posture, loss of response to stimuli and loss of >20% body weight were reached. After euthanasia, the lung and spleen were removed and homogenized in 1 ml 0.9% sterile saline. Serial dilutions of tissue homogenates were plated on LB agar and bacterial CFU were counted after 2 days of incubation at 37°C.

For the effects of treatment on organ bacterial burden and dissemination, 30 mice were divided into 6 treatment groups (n = 5/group): TA (50 mg/kg) suspended in corn oil given via oral gavage, CZ (200 mg/kg and 25 mg/kg) dissolved in sterile PBS (pH 7.4) given subcutaneously, or co-treated with TA (50 mg/kg) at a sub-therapeutic dose of CZ (25 mg/kg), untreated, or vehicle control (corn oil) via oral gavage. Once the untreated infected control group showed signs of morbidity (typically around 60 hours post infection), mice were euthanized, and the lung and spleen from 3 mice (selected randomly) from each group were homogenized and plated for bacterial load determination.

For the survival study, mice were divided into 3 treatment groups (n = 5/group): 25 mg/kg CZ administered subcutaneously, 50 mg/kg TA administered via oral gavage in corn oil, and a co-treatment of 25 mg/kg CZ and 50 mg/kg TA. After the treatment, mice were monitored daily for signs of mortality for up the 37 days post-infection.

### 2.8 Statistical Analysis

Statistical analyses was performed using Prism 6.0 software (Graphpad). Log-rank Mantel-Cox analysis was conducted for survival curves. All other data were analyzed using a one-way or two-way ANOVA followed by the Bonferroni post-test to determine statistical differences between groups or a two-tailed t-test for experiments with less than three groups. p<0.05 was considered statistically significant.

## Results

### 3.1 TA Treatment Increases Cell Viability In-vitro

**Fig 1:**
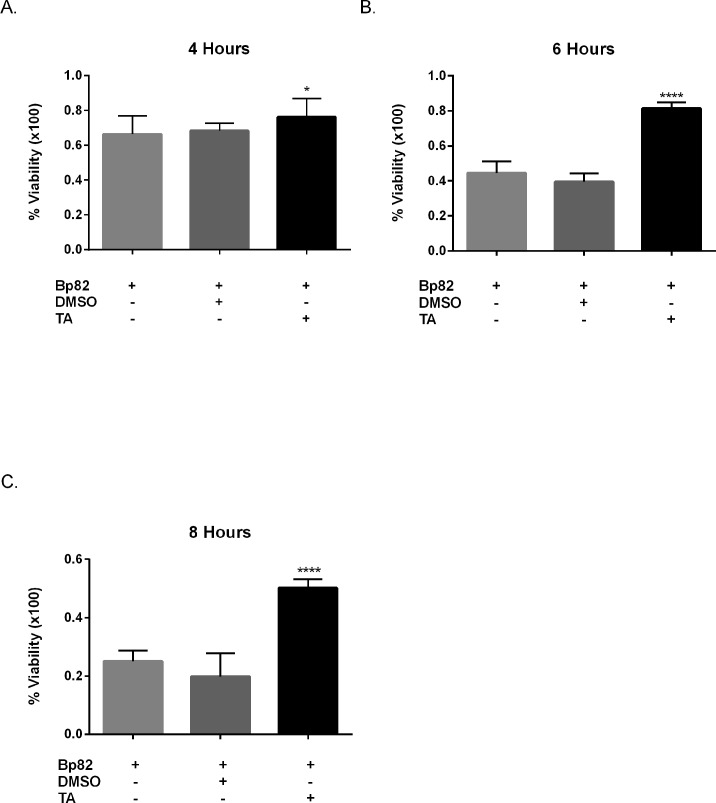
Effect of immune modulation on RAW 264.7 cell viability when infected with Bp82. For each time point, cells were pretreated for 30 minutes with 100μM TA, 0.01% DMSO or were untreated. Infection groups were then infected with a Bp82 MOI of 5. Cell viability was determined via optical absorbance at 570 nm utilizing the crystal violet method. Each treatment was normalized to uninfected/untreated control for each time point. The experiment was conducted in biological triplicate with ten technical replicates. Biological replicates were compared to the vehicle control via 2-way ANOVA with Bonferroni’s post-hoc test for significance. A small significance was observed at 4 hours with TA treatment (A) where both 6 hours (B) and 8 hours (C) revealed a dramatic increase in cell viability with TA treatment. Error indicated SEM *p<0.05, ****p<0.0001.

To determine if treatment with TA resulted in increased cell viability *in-vitro*, pretreated RAW 264.7 cells were infected with Bp82 at an MOI of 5 and cell viability was monitored over 4, 6, and 8 hours. Fig 1 reveals that Bp82 infection resulted in 44% reduction in cell viability after 4 hours, 66% after 6 hours, and 75% after 8 hours. RAW 264.7 cell cultures pretreated with 100μM TA revealed a reduction in cytotoxicity induced by Bp82 by 8% at 4 hours, by 42% at 6 hours and 30% at 8 hours when compared to the vehicle control group. Experimentation after 8 hours was not conducted as greater that 75% cytotoxicity was shown in later time points.

### 3.2 Minimum Inhibitory Concentrations

100 μM TA is a significant treatment, as treatments of this magnitude have shown to limit cell growth and cause cell death in colon cancer cells via activation of apoptosis pathways [9]. Therefore, we needed to confirm TA did not possess an inherent chemical nature that affected the bacterial growth and proliferation of Bp82. The MIC of TA exceeds 489 μM (Table 1), which exceeds the treatment doses used in this study. This suggests that the ability of TA in enhancing cell viability is a result of the immune modulatory properties of TA. Additionally, the MIC of DMSO was confirmed to be greater than 10%, confirming that the 0.01% vehicle concentration used in this study does not affect bacterial growth and proliferation. The MIC of CZ was used as an experimental control. The MIC of CZ fell within previously published results [16].

**Table 1:** MIC Determinations.

Drug	Bp 82 MIC
TA	>489 μM
CZ	7.31 μM
DMSO	> 10%

### 3.3 TA Inhibits Inflammatory Pathways Induced by Bp82

**Fig 2:**
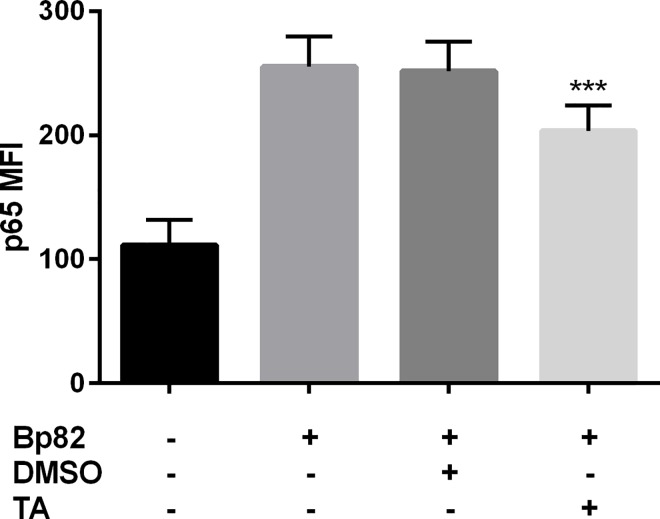
The effect of immune modulation on RAW 264.7 NF-ĸB p65 expression when infected with Bp82 after 90 minutes of infection. Cells were pretreated with 100 μM TA or 0.01% DMSO for 30 minutes along with an uninfected and untreated control. NF-ĸB p65 levels were determined flow cytometry using the Mean Channel Fluorescence (MCF) over 5,000 events from intact non-aggregated cells (see S1 Fig). Significance was determined by comparing to the vehicle control, where TA treatment was able to suppress p65 expression significantly. Data is representative of three biological replicates run in duplicate. Significance was determined via 2-way ANOVA with Bonferroni’s post-hoc test for significance. Error indicates SEM. ***p<0.001, ****p<0.0001.

A combination of flow cytometry and western blotting were used to confirm Bp82 activation of the NF-ĸB pathway. Infection results in a 2.5 fold increase in p65 mean channel fluorescence (MCF) in RAW 264.7 cells (Fig 2) and pretreatment with TA reduced the MCF by 20% when compared to the DMSO control. This suggests that TA is able to significantly reduce p65 levels in Bp82 infected RAW 264.7 cells. S3 Fig confirms the effects of TA on p65 via western blot. This is consistent with previously published work as TA was shown to reduced p65 levels in LPS activated RAW 264.7 cells [10].

**Fig 3:**
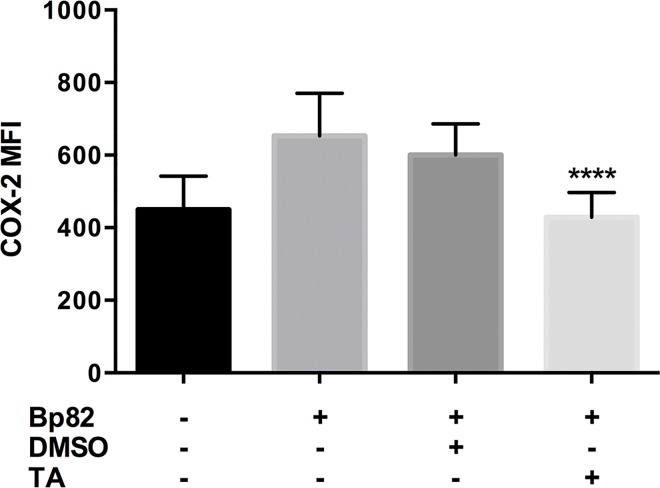
The effect of immune modulation on RAW 264.7 COX-2 expression when infected with Bp82 after 6 hours of infection. Cells were pretreated with 100 μM TA or 0.01% DMSO for 30 minutes along with an uninfected and untreated control. COX-2 levels were determined flow cytometry using the Mean Channel Fluorescence (MCF) over 5,000 events from intact non-aggregated cells (see S1 Fig). Significance was determined by comparing to the vehicle control, where TA treatment was able to suppress COX-2 expression significantly. Data is representative of three biological replicates run in duplicate. Significance was determined via 2-way ANOVA with Bonferroni’s post-hoc test for significance. Error indicates SEM. **p<0.01, ****p<0.0001.

Infection with Bp82 resulted in a ~1.5-fold increase in COX-2 MCF from uninfected groups as presented in Fig 3. We also determined that pretreatment of RAW 264.7 cells pre-treated with 100 μM TA and infected with Bp82 resulted in a ~30% reduction in COX-2 MCF versus the vehicle control group. S5 Fig offers visual representation of this reduction in COX-2 via immunofluorescence.

**Fig 4:**
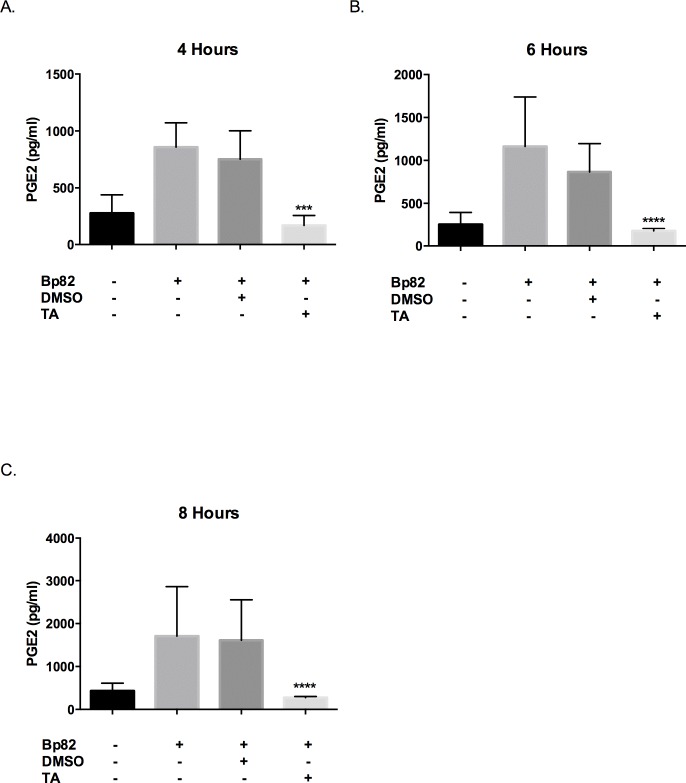
Effect of TA treatment on PGE2 levels during an infection of RAW 264.7 cells with Bp82. For each time point, cells were pretreated for 30 minutes with 100μM TA, 0.01% DMSO or were untreated. Infection groups were then infected with a Bp82 MOI of 5. PGE2 levels were determined via ELISA (R&D Systems) in accordance with the manufacturer’s instructions. The results represent three independent experiments plated in duplicate and were compared to the vehicle control via 2-way ANOVA with Bonferroni’s post-hoc test for significance. Error indicates SEM. Treatment with TA revealed a reduction in PGE2 levels at all time points (A-C). There is no significance between the uninfected groups and the TA treated groups. **p<0.01, ****p<0.0001.

It has been shown that PGE2 plays an important role during infection and its suppression is a possible therapeutic strategy [18]. Additionally, it has been shown that *B*. *thailandensis* infection results in increased production of PGE2, suggesting that PGE2 is necessary for the bacteria’s intracellular survival [5]. Our next goal was to confirm that Bp82 upregulates the production of PGE2 in RAW 264.7 cells and pretreatment with 100 μM TA reduces PGE2 production. Indeed, Bp82 infection resulted in 3-fold increase in supernatant PGE2 concentration after 4 hours of infection, and over a 4 fold increase in supernatant concentration at 6 and 8 hours. TA effectively reduced PGE2 levels by over 4-fold at all time points when compared to the vehicle control (Fig 4). Additionally, it would appear that pretreatment with TA reduced the PGE2 concentration to levels lower than the uninfected control, however, this was not significant.

### 3.4 Co-treatment of TA with Sub-Therapeutic Ceftazidime reduces *B*. *pseudomallei* Burden and Increases Survival in infected Mice

**Fig 5:**
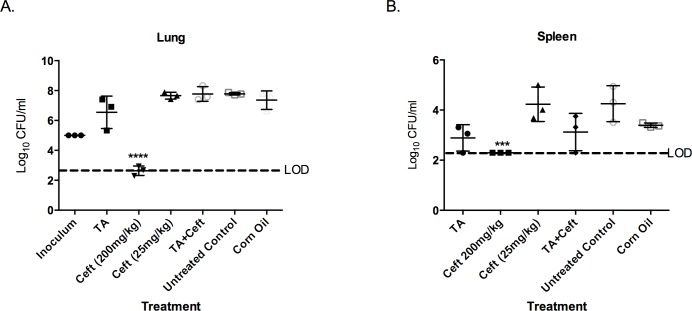
Comparison of the CFU burdens in the lung and spleen after a lethal pulmonary infection after 60 hours. BALB/c mice (n = 5 per group) were infected with 2 X 10³ CFU (~3 X LD50) *of B*. *pseudomallei* 1026b intranasally. Three hours post-infection treatments were administered and then again daily for two consecutive days. Organ homogenates from 3 mice were plated in serial dilution in duplicate and compared to the corn oil treatment group (vehicle control) for significance using a 1-Way ANOVA with Bonferroni’s post-hoc test for significance. CZ was the only treatment able to significantly reduce CFU burden in the lung and spleen (A-B). However, there is a noticeable decrease in spleen bacterial burden in both groups treated with TA. The inoculum dose is included in the lung data (A) for comparison. LOD indicates the limit of detection using the logarithmic scale. Error indicates SD. ***p<0.001, ****p<0.0001.

The only treatment successful at significantly reducing the bacterial burden in both the lung and spleen was 200 mg/kg CZ (Fig 5). This confirms 200 mg/kg CZ administered subcutaneously is effective at reducing bacterial burden as shown in previous studies [19]. To assess if immune-modulation can potentiate the efficacy of CZ against an acute *B*. *pseudomallei* infection in the murine model, TA was co-administered with a sub-therapeutic dose of CZ. This data also indicates that 25 mg/kg CZ was ineffective at reducing the bacterial burden in the lung and spleen.

**Fig 6:**
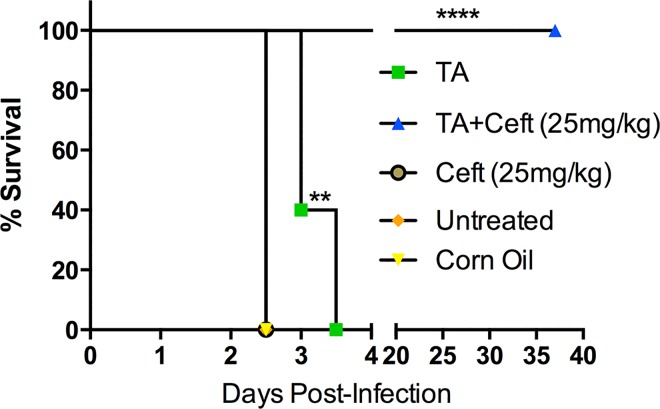
TA treatment and co-treatment of TA with a sub-therapeutic antibiotic treatment provides significant protection against lethal pulmonary melioidosis. BALB/c mice (n = 5 per group) were infected with 2 X 10³ CFU (~3 X LD50) of *B*. *pseudomallei* 1026b intranasal. Three hours post-infection treatments were administered, then again daily for two consecutive days totaling three treatments. Survival was monitored for 37 days. Statistical significance was determined via the Mantel-Cox test. **p<0.01, ****p<0.0001.

**Fig 7:**
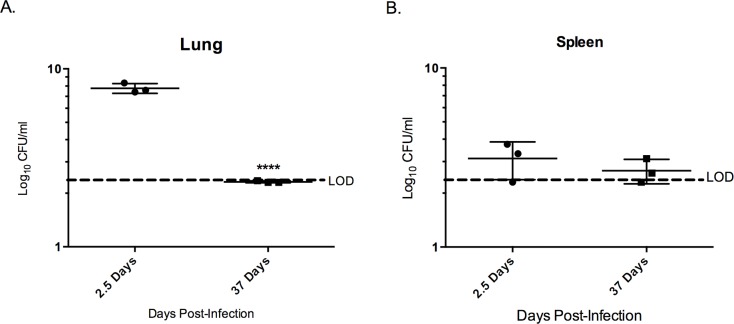
Co-treatment of TA treatment with sub-therapeutic CZ treatment may present a novel treatment for melioidosis. After 37 days post-infection without indication of mortality from 3 X LD50 inoculation with *B*. *pseudomallei* 1026b with only three consecutive treatments in two days, the mice were sacrificed to determine the bacterial burden in the lung and spleen. These organs were removed, homogenized and plated in serial dilutions in duplicate. This data was then compared to the same treatment group at 2.5 days post-infection to determine if there was a significant reduction in bacterial burden over time. The lung (A) showed significant reduction in bacterial burden in the 35 days after treatment was terminated. The spleen did experience a reduction in CFU burden, however, this was determined not to be significant. Samples were analyzed unpaired two-tailed t-test for significance. Error indicates SD. ***p<0.001, ****<0.0001.

In TA-treated mice there was a 40% increase in survival time for 40% of the group (Fig 6). Co-treated TA/CZ group had 100% survival until 37 days post infection (pre-determined study endpoint). After 37 days, bacterial burden was assessed in the lung and spleen and compared to burden assessed at day 2.5 (Fig 7). Notably, the lung showed a reduction in bacterial burden of ~5x10^7^ CFU/ml and the spleen a slight reduction of ~1 x 10^3^ CFU/ml between 2.5 and 37 days post infection.

## Discussion

Combating the problem of antibacterial resistance is a global responsibility. This is particularly relevant for B. *pseudomallei* due to its ability to efflux many antibiotics [20]. Novel conjunctive therapies should be investigated to reduce the current antibacterial treatment regimes. Immunomodulation utilizing COX-2 inhibition during *B*. *pseudomallei* infection may prove an effective strategy to increase antibacterial effectiveness and treat the disease.

We corroborated previous studies by Asakrah et al [5] on cytotoxicity and the important role of COX-2 and PGE2 during melioidosis. These findings were expanded upon by investigating the effects of TA treatment on the NF-ĸB pathway during infection. We were able to mimic similar treatment outcomes using TA, which is approved for human use in certain areas around the globe [7]. Most importantly, we showed that the conjunctive treatment of immunomodulation with sub-therapeutic antibiotics significantly increased survival outcome and decreased organ bacterial burdens.

PGE2 has been linked to the regulation of a number homeostatic biological functions. Of greatest relevance here is that its involvement in initiating the classic signs of inflammation including redness and swelling due to PGE2-mediated arterial dilatation and increased vascular permeability and pain from PGE2 acting on sensory neurons [21] Additionally, all of the aforementioned mechanisms can lead to tissue damage. This compromised tissue provides an opportune environment for bacterial proliferation. Limiting inflammatory damage during bacterial infections has been shown to be an effective therapeutic strategy [6]. Our *in-vitro* studies revealed that limiting COX-2 induction and PGE2 production using TA translated to a significant increase is cell viability in mouse macrophage-like RAW 264.7 cells. This further confirms the results published by Asakrah et al [5]. Furthermore, pretreatment with TA was not only able to inhibit COX-2, as shown by the reduction of PGE2 production, but treatment also limited COX-2 induction. This may be linked to effects of TA on NF-ĸB and the interaction between the two inflammatory pathways.

NF-ĸB may prove an important inflammatory target during *B*. *pseudomallei* infection as it is believed to activate during infection and lead to the translation of many pro-inflammatory cytokines [3]. These cytokines amplify the systemic inflammatory response, often times by inducing COX-2 and resulting in continued production of PGE2 and other prostaglandins. PGE2 can have a positive feedback loop with NF-ĸB by increasing its transactivation and enhancing the production of pro-inflammatory cytokines [22]. Previous studies indicate TA is effective at reducing NF-ĸB activation in stimulated RAW 264.7 cells [10], but it is unclear whether this is linked to the PGE2/NF-ĸB relationship. Here, we show that treatment with TA reduces Nf-ĸB p65 during *B*. *pseudomallei* infection.

Immunomodulation may be necessary for comprehensive treatment of bacterial infections, particularly to combat bacterial resistance [6]. Our *in-vivo* studies reveal that immune modulation with orally administered TA is effective at increasing survival outcome in BALB/c mice. This is consistent with the findings of Asakrah et al [5]; although those studies were conducted using NS-398, an experimental COX-2 inhibitor given intraperitoneally. TA treatment alone did not show the same efficacy as NS-398 by Asakrah et al [5] but this may be due to the present dose being too low. More research is needed to determine if a larger dose of TA affords greater protection from a lethal *B*. *pseudomallei* pulmonary challenge.

Co-treatment of TA and CZ significantly increased survival outcome, and contributed to a nearly complete bacterial clearance after 37 days post infection. This suggests that the immunomodulation activity of TA allows for the exploitation and enhancement of the therapeutic benefit of CZ.

To the best of our knowledge, this is the first study indicating that immune modulation with orally-administered TA enhances the therapeutic benefit of antibiotic treatment during pulmonary melioidosis. The increased survival outcome resulting from the reduction of PGE2 production during bacterial infections through COX-2 inhibition and reduction in NF-ĸB activation has profound implications. PGE2 is produced during a number of lethal bacterial infections (i.e *Francisella tularesis* [23]) and opportunistic bacterial infections (*Pseudomonas aeruginosa* and *Staphylococcus aereus*) for which antibacterial treatment is complex due to antibacterial resistance [24] [25]. Proving the efficacy of immunotherapy using commercially available, orally administered TA in combination with sub-therapeutic antibiotic treatment during melioidosis in BALB/c mice warrants further investigation in other animal models of melioidosis. Such experiments may further reveal the effectiveness of TA and other NSAIDS across a wide range of bacterial infections. While this may have a profound impact on treatment of human bacterial infections, additional animal studies are needed to ensure efficacy of this treatment strategy before human clinical trials.

## Supporting Information

S1 Data Dose Response. RAW 264.7 cells were pretreated with 10 μM, 20 μM, 50 μM, 100 μM, 150 μM and 200 μM TA and 0.01% DMSO for 30 minutes and infected with Bp82 at an MOI of 5. Cell viability was determined via optical absorbance at 570nm utilizing the crystal violet method. Each treatment was normalized to uninfected/untreated control for each time point. The experiment was conducted in biological triplicate with ten technical replicates. Biological replicates were compared to the vehicle control via 2-way ANOVA with Bonferroni’s post-hoc test for significance. 100 μM was chosen for all further *in vitro* based on its consistent ability to increase cell viability at all time points.
(TIF)

S1 Fig Flow cytometry gating of intact cells. RAW 264.7 cells were gated on a linear forward-scatter vs. logarithmic side-scatter plot.
(TIF)

S2 Fig Eliminating doublets from analyzed population. To eliminate doublets from the population analyzed for MCF a pulse-width vs logarithmic mean intensity plot was used to gate on the singlet population. This is a representative image for COX-2 analysis. This population was used to determine the MCF.
(TIF)

S3 Fig Histogram confirming unstained control. This is a representative image of the histogram shifts that occur when comparing the uninfected/unstained, uninfected/stained, and Bp82 infected/stained.
(TIF)

S4 Fig Confirmation of NF-ĸB activity with infection of treatment. A representative western blot image of the p65 activity with infection and treatment after 90 minutes of infection. Infection of RAW 264.7 with Bp82 resulted in an increase in p65 expression, and pre-treatment with TA was able to significantly reduce this expression. Data is representative of duplicate experiments.
(TIF)

S5 Fig Confirmation of COX-2 expression with infection and treatment. A representative immunofluorescence image with infection and treatment after 6 hours of infection. On the left, DAPI was used for the nuclear counter stain (blue).Infection of RAW 264.7 with Bp82 resulted in induction of COX-2 (red) and pre-treatment with TA significantly reduced COX-2 expression. The right column displayed the DAPI and COX-2 images merged.
(TIF)
