# Supplementary figures and images for: Immune Modulation as an Effective Adjunct Post-exposure Therapeutic for B. pseudomallei
Source: PLoS Negl Trop Dis. 2016 Oct 28;10(10):e0005065. doi: 10.1371/journal.pntd.0005065 (PMC5085046; doi:10.1371/journal.pntd.0005065)

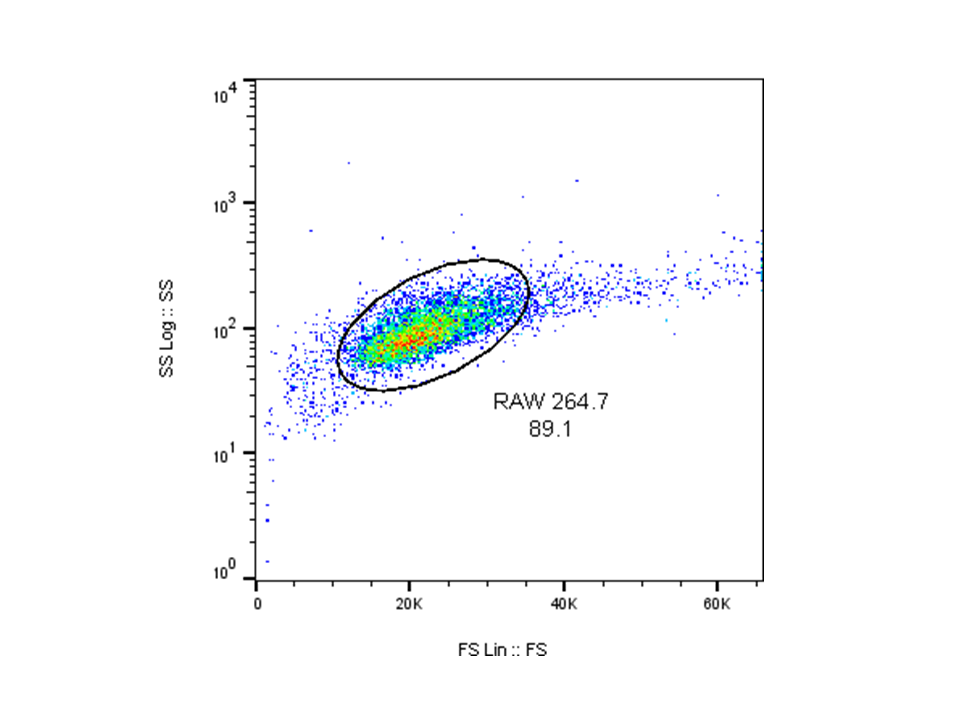

Supplement: S1 Fig — RAW 264.7 cells were gated on a linear forward-scatter vs. logarithmic side-scatter plot. (TIF) [file pntd.0005065.s002.tif]

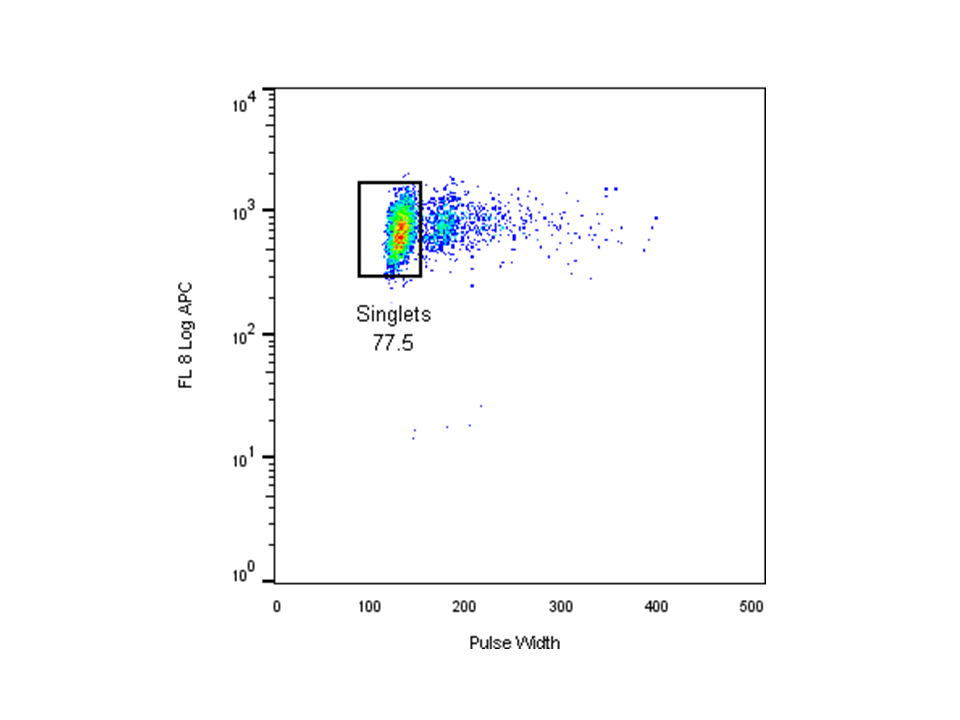

Supplement: S2 Fig — To eliminate doublets from the population analyzed for MCF a pulse-width vs logarithmic mean intensity plot was used to gate on the singlet population. This is a representative image for COX-2 analysis. This population was used to determine the MCF. (TIF) [file pntd.0005065.s003.tif]

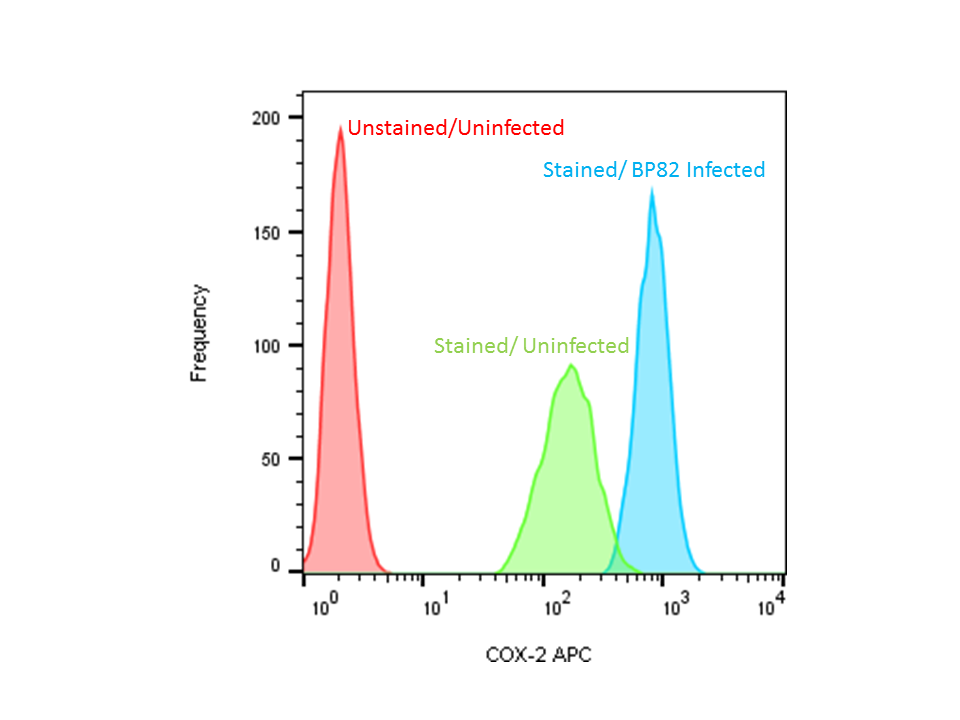

Supplement: S3 Fig — This is a representative image of the histogram shifts that occur when comparing the uninfected/unstained, uninfected/stained, and Bp82 infected/stained. (TIF) [file pntd.0005065.s004.tif]

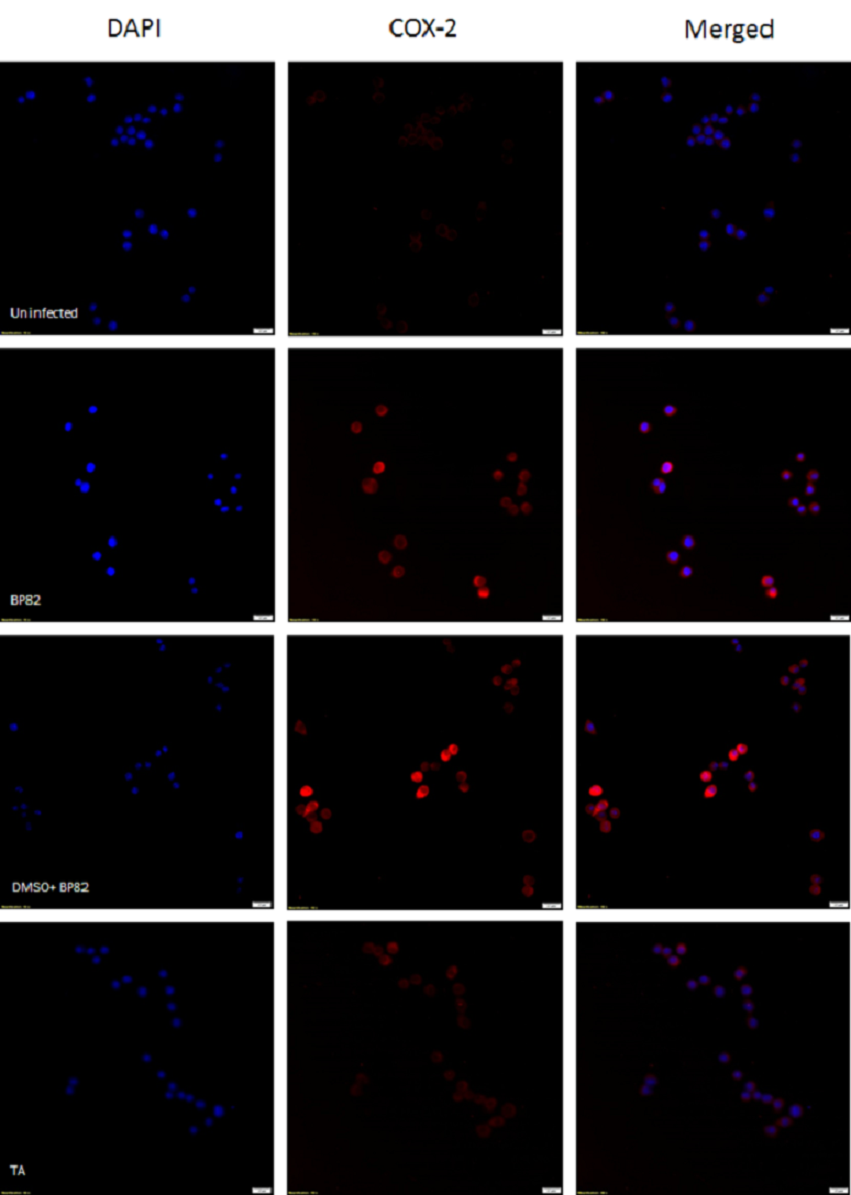

Supplement: S5 Fig — A representative immunofluorescence image with infection and treatment after 6 hours of infection. On the left, DAPI was used for the nuclear counter stain (blue).Infection of RAW 264.7 with Bp82 resulted in induction of COX-2 (red) and pre-treatment with TA significantly reduced COX-2 expression. The right column displayed the DAPI and COX-2 images merged. (TIF) [file pntd.0005065.s006.tif]
